# Intra-arterial pulse wave analysis during thrombectomy for the assessment of collateral status – A feasibility study

**DOI:** 10.1371/journal.pone.0210572

**Published:** 2019-01-10

**Authors:** Johanna Sandmann, Thorsten Sichtermann, Franziska Sonja Müschenich, Hadi Nasri, Sarah Heringer, Anastasios Mpotsaris, Martin Kramer, Martin Wiesmann, Omid Nikoubashman

**Affiliations:** 1 Department of Diagnostic and Interventional Neuroradiology, University Hospital, RWTH Aachen University, Aachen, Germany; 2 Department of Veterinary Clinical Sciences, Small Animal Clinic, Justus-Liebig-University, Gießen, Germany; Medical University Innsbruck, AUSTRIA

## Abstract

**Purpose:**

Knowledge of the collateralization of an occluded vessel is important for the risk-benefit analysis of difficult revascularization maneuvers during mechanical thrombectomy. If the territory behind a clot is well perfused, one could desist from performing a risky thrombectomy maneuver. The arterial pulse pressure curve may serve as an indicator for the collateralization of an occluded target vessel. We investigated the feasibility of arterial pulse measurements with a standard microcatheter.

**Methods:**

We measured the intra-arterial blood pressure proximal and distal to the clot in 40 thrombectomy maneuvers in a porcine stroke model. We used a microcatheter (Trevo Pro 18, Stryker, Kalamazoo, CA, USA), a pressure transducer (MEMSCAP SP844), an AdInstruments Powerlab 16/35 workstation, and LabChart 8 Software (AdInstruments, Dunedin, New Zealand).

**Results:**

Median arterial blood pressure proximal and distal to the clot was 96.0 mmHg (IQR, 23.8 mmHg) and 47.5 mmHg (IQR, 43.5 mmHg), respectively (p < .001). The median difference between systolic maximum and diastolic minimum proximal and distal to the clot decreased significantly from 1.8 mmHg (IQR, 3.6 mmHg) to 0.0 mmHg (IQR, 0.5 mmHg) (p < .001). There was loss of the curve in 26 of 40 cases and loss of pressure in 23 of 40 cases (p = .008). There was no significant correlation between vessel diameter and either loss of the pulse pressure curve (p = .20) or overall pressure loss (p = .31).

**Conclusion:**

It is possible to measure the pulse pressure proximal and distal to the clot with a standard microcatheter used during mechanical thrombectomy.

## Introduction

The collateral circulation has an important impact on clinical outcome of stroke patients because collaterals sustain blood flow in the stroke penumbra [1]. The collateral status can be estimated with CT perfusion and CT angiography [2, 3]. Sorimachi et al. have also shown that it is possible to estimate the collateral status of a single vessel during endovascular stroke treatment [4, 5]. The authors compared the blood pressure gradient proximal and distal to the clot during intra-arterial thrombolysis and postulated that a pressure loss of at least 30 mmHg behind the clot reflects poor collateralization and is associated with worse clinical outcome. Knowledge of the collateralization of a target vessel may be a useful information for the risk-benefit analysis of difficult revascularization maneuvers: While the vast majority of thrombectomy maneuvers is considered to be safe, performing many thrombectomy maneuvers in small and/or curved distal branches may lead to vessel perforation [6]. Hence, one could desist from performing risky thrombectomy maneuvers in very distal or curved branches, if the territory behind a clot is collateralized well.

We believe that the pulse pressure of the arterial pulse wave may serve as an additional parameter for the estimation of the collateral status. We hypothesize that the typical arterial pulse curve with a systolic maximum and a diastolic minimum should remain preserved behind the clot if the occluded vessel is well collateralized. Poor collateralization on the contrary should lead to a loss of the pulse pressure of the arterial pulse curve. The aim of our study was to investigate the feasibility of arterial pulse measurements with a standard microcatheter used for mechanical thrombectomy and to assess whether loss of the arterial pulse pressure behind a clot can be measured. To investigate the feasibility of arterial pulse measurements with a standard microcatheter, we measured the arterial blood pressure during thrombectomy maneuvers in a porcine stroke model. Because the porcine stroke model does not allow quantification of collateralization, blood pressure loss and vessel diameter served as surrogate parameters for collateralization [7].

## Methods

### Animals

The measurements were performed in accordance with the (blinded) legislation governing animal studies following the “Guide for the Care and Use of Laboratory Animals” (National Research Council, 8th edition, 2011) and the “Directive 2010/63/EU on the Protection of Animals Used for Scientific Purposes” (EU Official Journal, 2010). Official permission was granted from the governmental animal care and use office (blinded).

Measurements were made in four female German Landrace swine (Gerd Heinrichs, Heinsberg-Karken, Deutschland; mean weight of 55.1kg ± 6.5kg, mean age of 5.3 months ± 0.5 month). The animals were housed under controlled environmental conditions (20°C ± 1°C, 12:12h light/dark cycle). Before starting the experiments, there was an acclimatization period of 2 weeks. Animals were kept in groups of 2 to 4 and received food and water ad libitum.

The animals received premedication by intramuscular injection of atropine (Atropinsulfat, B. Braun Melsungen AG, Melsungen, Germany), azaperone (Stresnil 40mg ad. us. vet.; Sanochemia Pharmazeutika AG, Neufeld, Austria) and ketamine (10% Ketavet ad. us. vet., Zoetis Deutschland GmbH, Berlin, Germany) [7]. Intubation was followed by mechanically ventilation with an oxygen-air mixture. Anaesthesia was maintained with continuous intravenous infusion with propofol (Propofol 2% MCT Fresenius; Fresenius Kabi Deutschland GmbH, Neuss, Germany). For analgesia fentanyl (Fentanyl-Janssen 0,5mg, Janssen-Cilag GmbH, Neuss, Germany) was continuously administered. Vital functions were monitored during the entire experiment. All animals were treated with ASS (Aspirin 500 mg) and heparin (3000 IU, Heparin-Natrium-5000; ratiopharm GmbH, Ulm, Germany) intravenously to prevent blood coagulation during interventions.

The narcotized animals were punctured at the femoral artery followed by insertion of an 8F sheath for further endovascular procedures. The experiments were acute trials, animals were euthanized by intravenous injection of 0.5- 1ml/kg body weight natrium-pentobarbital (Narcoren 16g/100ml; Merial GmbH, Hallbergmoos, Germany).

### Blood pressure measurement

First, we assessed the vasculature of the upper limbs via DSA. We then injected clots in the branches of the upper limb arteries following published procedures and we measured the diameters of the occluded vessels [8, 9]. We then measured the intra-arterial blood pressure 5–10 mm proximal and distal to the clot with a Trevo microcatheter (Trevo Pro 18, Stryker, Kalamazoo, CA, USA) using a pressure transducer (MEMSCAP SP844), an AdInstruments Powerlab 16/35 workstation, and LabChart 8 Software (AdInstruments, Dunedin, New Zealand). The pressure transducer was connected to the microcatheter via a standard 3-way valve (Discofix C; Braun, Melsungen, Germany) and tubing (Original-Perfusor line 150 cm; Braun). Continuous flush through the catheter was interrupted during measurements via the 3-way valve. First, we assessed the pressure immediately before the clot. Then, we passed the clot with the microcatheter (n = 46) or the microwire (n = 2) first and confirmed the correct position of the microcatheter with a superselective angiography series [6]. We then measured the pressure behind the clot. We measured the blood pressure until a stable value of blood pressure was reached, which took approximately 5 to 30 seconds distal to the clot. Afterwards, the microcatheter was pulled back through the clot to its proximal end and the blood pressure was measured again following the same procedure. Overall, we measured pressures in 48 cases.

### Data and statistical analysis

Blood pressure was recorded continuously with a sampling rate of 1000 Hz. For data analysis we used the MATLAB R2017a software (The Mathworks, Natick, USA). We applied a high pass filter (Butterworth, 3^rd^ order, cut-off frequency (f_C_) = 1 Hz) for suppressing signal wander induced by e.g. breathing and a third order low pass filter (Butterworth, 3^rd^ order, f_C_ = 40 Hz) for filtering high-frequency noise and movement artefacts.

**Fig 1:**
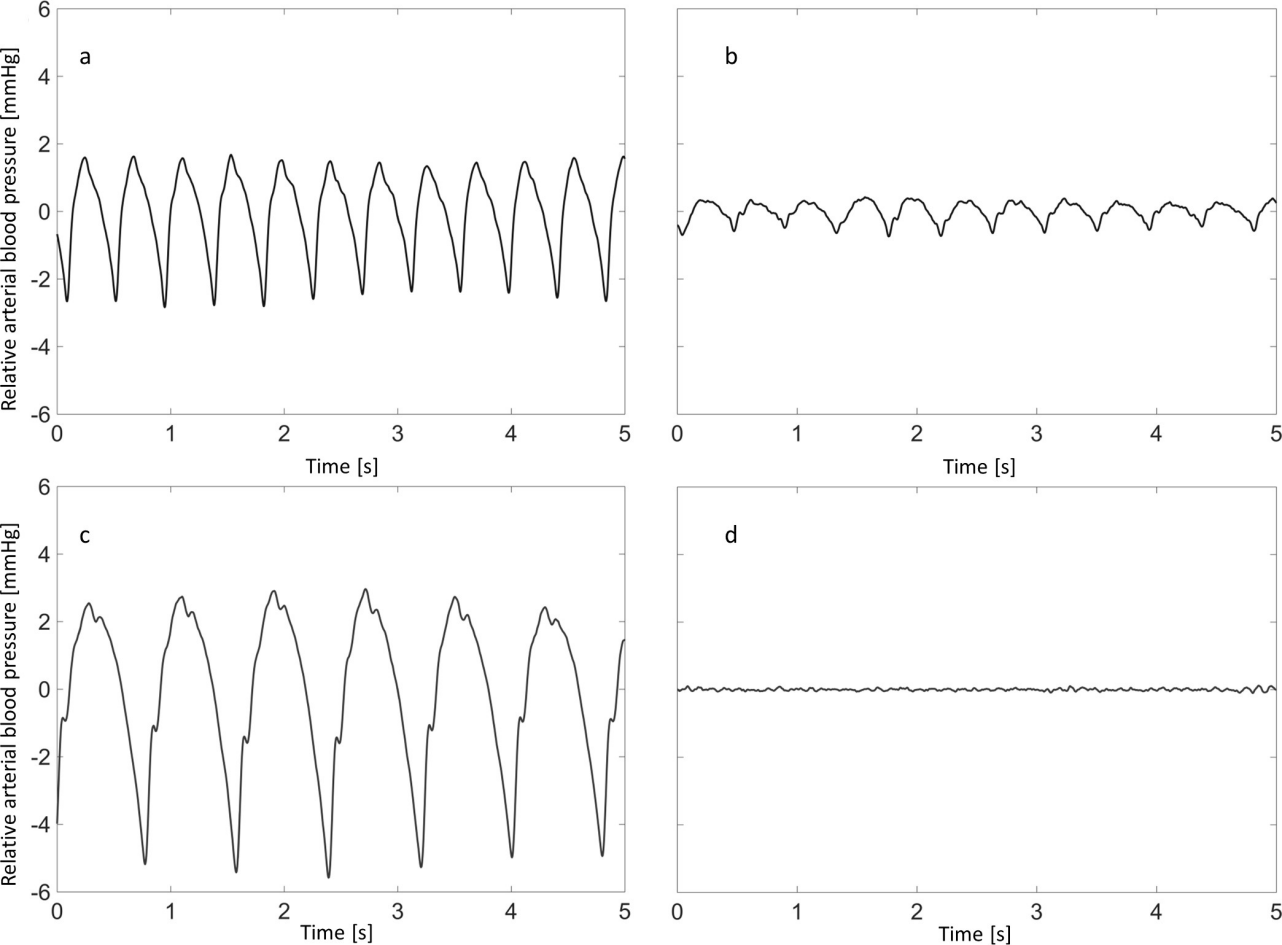
Arterial pulse wave proximal and distal to the clot. Upper row (a and b): case with maintained arterial pulse wave. Pressure curves before (a) and behind (b) the clot. Mean arterial pressure and pulse pressure before (a) and behind (b) the clot are 100 mmHg and 86 mmHg and 4.4 mmHg and 0.5 mmHg, respectively. Lower row (c and d): case with loss of arterial pulse wave. Pressure curves before (c) and behind (d) the clot. Mean arterial pressure and pulse pressure before (c) and behind (d) the clot are 98 mmHg and 10 mmHg and 7.3 mmHg and 0 mmHg, respectively.

Blood pressure was defined as the average of all values acquired over a period of 5 seconds. The arterial pulse pressure was defined as the magnitude of the difference between the systolic maximum and the diastolic minimum blood pressure averaged over a period of 5 seconds. Whenever the typical arterial pulse curve with its systolic maximum and diastolic minimum was not detectable, this was noted as a loss of the pulse curve (Fig 1). In accordance with Sorimachi et al. we defined pressure loss as falling below the cut-off value at 30 mmHg [4, 5]. As an alternative definition of pressure loss, we postulated that there was pressure loss whenever the arterial blood pressure behind the clot was below two standard deviations of the blood pressure proximal to the clot. As our stroke model does not allow quantifying the collateralization of vessels, we hypothesized that large arteries are collateralized better than small arteries and calculated the relationship between vessel size and pressure.

Continuous parametric variables are presented as mean ± standard deviation, non-parametric variables as median with interquartile range. After testing for normal data distribution with the Shapiro-Wilk test, Mann-Whitney U tests were used to determine whether there is a difference in either blood pressure or arterial pulse wave proximal and distal to the clot, respectively. We also examined the relationship between vessel diameter and loss of blood pressure (pressure loss and pulse pressure loss) by cross-tabulation using Pearson’s χ2 tests and Pearson’s correlation coefficient. Values with an α level < .05 were considered as statistically significant. All statistical analyses were calculated in SPSS V.23 software (IBM, Armonk, New York, USA).

## Results

Eight of 48 measurements were excluded, leaving 40 maneuvers for statistical analysis: in three cases no measurements were possible because the microcatheter was clogged. In the remaining five cases, the clot was only partly occluding the vessel, hereby equaling the pressure before and behind the clot. In the 40 included cases, average vessel diameters before and behind the clot were 2.5±0.8 mm (range, 1.2–5.9 mm) and 2.3±1.0 mm (range, 1.3–5.4 mm), respectively. Median arterial blood pressure proximal and distal to the clot were 96.0 mmHg (IQR, 23.8 mmHg) and 47.5 mmHg (IQR, 43.5 mmHg), respectively (p < .001). The median pulse pressure proximal and distal to the clot decreased significantly from 1.8 mmHg (IQR, 3.6 mmHg) to 0.0 mmHg (IQR, 0.6 mmHg) (p < .001). There was a significant correlation between high blood pressure and high pulse pressure before the clot on the one side and low blood pressure and low pulse pressure behind the clot on the other (p < .001). There was a loss of the arterial pulse pressure in 24 of 40 cases. There was pressure loss as defined above and as defined by Sorimachi et al. (cut off value = 30 mmHg) in 23 of 40 cases and in 24 of 40 cases, respectively. The correlation between pulse pressure loss and pressure loss as defined above and as defined by Sorimachi et al. was significant (p = .006 and p = .03, respectively). There was no significant correlation between vessel diameter and either pulse pressure loss (p = .20) or pressure loss as defined by Sorimachi et al. (p = .20) or us (p = .31).

## Discussion

Ever since mechanical stroke treatment has been established as a standard treatment method, neurointerventional stroke research has shifted to finding the optimal treatment technique [10–15]. A further focus that is emerging is the issue of correct patient selection. Knowledge of the collateralization of an occluded target vessel may help to facilitate the decision whether the benefits outweigh the risks of a complicated thrombectomy maneuver; for instance one could desist from performing further thrombectomy maneuvers after several futile catheterization and thrombectomy attempts in distant branches, if the territory behind the clot is collateralized well. Another interesting indication could be acute carotid artery stenting if it is unclear whether carotid artery occlusion is acute or chronic. Collateral flow indicating a pre-existing chronic occlusion could be a helpful tool for the correct differentiation between acute and chronic occlusions.

We hypothesized that the arterial pulse wave provides useful information about the collateralization of a target vessel during mechanical thrombectomy. In this study, we have shown that it is possible to measure the pulse pressure curve proximal and distal to the clot with standard microcatheters used during mechanical thrombectomy. As expected, there was loss of the arterial pulse pressure curve in some cases. We also found that this loss of the pulse pressure correlated significantly with pressure loss. We believe that loss of the pulse pressure may reflect collateralization better than pressure loss, because predefined cut-offs may not reflect the inter- and intra-individual variability of baseline values. Unfortunately, the porcine stroke model does not allow the quantification of collateralization. Vessel diameter as a loose surrogate for collateralization did neither significantly correlate with pressure loss nor with pulse pressure loss. This is why better stroke models or in-patient data are needed to investigate this issue more thoroughly [16, 17].

### Limitations

The porcine stroke model in the upper limbs does not allow the quantification of collateral status and is therefore one of the major limitations of our study. Nonetheless, our results show that pressure pulse measurements are feasible and may serve as a foundation for further studies. A further limitation is the fact that we conducted our experiments with only one microcatheter, as mechanical properties such as the rigidity of the catheter may have an impact on our results [18]. Another difficulty was the choice of a suitable high and low pass filter. For baseline wander removal we used a Butterworth high pass filter which is both computationally fast and comparably accurate to more complex filter models [19]. Due to the simple filter design it will be easy to expand this method into real time analysis applications. We obtained the best results in terms of signal intensity and suppression of baseline wander at a cut off frequency of 1 Hz. Nevertheless, it was not possible to remove interfering signals completely (Fig 1). We also selected a suitable cut-off frequency for the low-pass filter by trial and error to suppress noise without removing the desired signal. Frequencies of 40 Hz or higher were removed by the filter.

## Conclusions

We have shown that it is possible to measure the pulse pressure proximal and distal to the clot with standard microcatheters used during mechanical thrombectomy. Knowledge of the arterial pulse pressure may serve as an indicator for the collateralization of an occluded target vessel, which in turn may help to facilitate the decision whether the benefits outweigh the risks of a complicated thrombectomy maneuver.

## Supporting information

S1 Checklist NC3Rs ARRIVE guidelines checklist. (PDF)
